# Prevalence of Non-Daily Teeth Cleaning and Its Associated Factors Among Adult Population in Rwanda

**DOI:** 10.3390/ijerph22071005

**Published:** 2025-06-26

**Authors:** Fabrice Iradukunda, Irene Bagahirwa, Bakang Percy Tlhaloganyang, Amparo Elena Gordillo-Tobar, Clarisse Musanabaganwa, Christian Nsanzabaganwa, Gad Nshimiyimana, Sincere Josue Ukuri, Jean Claude Habineza, Joel Gasana, Pacifique Igiraneza, Venantie Umuhoza, Violette Uwamungu, Alberto Barcelo, Francois Uwinkindi, Claude Mambo Muvunyi

**Affiliations:** 1 Non Communicable Disease, Rwanda Biomedical Center, Kigali P.O. Box 7162, Rwanda; iradukundafabrice13@gmail.com (F.I.); irene.bagahirwa2013@gmail.com (I.B.); clarisse.musanabaganwa@rbc.gov.rw (C.M.); nsanzechriss@gmail.com (C.N.); gad.nshimiyimana@rbc.gov.rw (G.N.); ukuri13@gmail.com (S.J.U.); jeanclaudehabineza@gmail.com (J.C.H.); joel.gasana@rbc.gov.rw (J.G.); igiranezapaci4@gmail.com (P.I.); venantieumuhoza2@gmail.com (V.U.); francois.uwinkindi@rbc.gov.rw (F.U.); claude.muvunyi@rbc.gov.rw (C.M.M.); 2 Health, Nutrition, and Population, World Bank Group, Washington, DC 20433, USA; agordillotobar@worldbank.org; 3 Educational Development and Quality Centre, University of Global Health Equity, Kigali P.O. Box 6955, Rwanda; violettewamungu@gmail.com; 4 Department of Public Health Sciences Research, University of Miami, Miami, FL 33136, USA; abarcelo@med.miami.edu

**Keywords:** daily teeth cleaning, associated factors, Rwanda

## Abstract

Oral hygiene practices are vital for maintaining health, yet many adults do not engage in daily teeth cleaning. This study aimed to assess the prevalence and determinants of non-daily teeth cleaning among adults in Rwanda using data from the 2022 Rwanda Non-Communicable Diseases (NCD) STEPS Survey which used a cross-sectional design and multistage cluster sampling. Weighted prevalence estimates and logistic regression models were used to examine associations between non-daily teeth cleaning and key demographic, socioeconomic, behavioral, and oral health factors. The prevalence of non-daily teeth cleaning was 33.1% (95% CI: 31.0–35.2). Rural residence (AOR = 2.5, 95% CI: 1.5–4.1), lower education (AOR = 0.3, 95% CI: 0.2–0.6), lower income (AOR = 2.0, 95% CI: 1.3–3.2), and not using toothpaste (AOR = 1.3, 95% CI: 1.0–1.7) were significantly associated with increased odds of non-daily teeth cleaning. These findings underscore the need for targeted oral health promotion strategies that address socioeconomic disparities and improve access to affordable hygiene products.

## 1. Introduction

Oral hygiene is a crucial component of general health, influencing not only the risk of dental caries and periodontal diseases but also systemic conditions such as cardiovascular disease and diabetes [1,2,3,4]. Poor oral health can result in tooth loss, infection, pain, impaired function, and diminished quality of life [1,2,3,4]. Despite being largely preventable, oral diseases remain widespread, with global estimates indicating that untreated caries affects over 3.5 billion people and accounts for significant health system burdens, particularly in low- and middle-income countries [3,5].

In Rwanda, oral health challenges are substantial, with caries experience reported in 64.9% of the population and over half (54.3%) of these cases untreated [6,7]. The 2022 NCD STEPS survey found that although 67% of adults clean their teeth daily, only 19.3% brush twice daily as recommended [8]. Additionally, 57% have never received dental care, and only 11.5% visited a dentist in the past year, mostly due to acute issues [8]. Several studies have confirmed that poor oral hygiene behaviors in Rwanda are associated with limited awareness, access issues, and socioeconomic disparities [1,2,9,10].

Factors such as low educational attainment, lack of knowledge about recommended practices, and rural residence are linked to poor oral hygiene [9,11,12]. A study from Muhima District Hospital showed that knowledge and awareness of twice-daily brushing significantly predicted better oral hygiene practices [13]. Studies from Kenya, Uganda, and Nigeria have highlighted similar challenges, with poor oral hygiene associated with limited resources, lack of awareness, and infrequent dental visits [14,15,16].

In addition to behavioral and knowledge-related barriers, structural challenges further compromise oral health in Rwanda. A recent study in Nyarugenge revealed that most public dental facilities lacked adequate equipment and staffing, with high patient-to-provider ratios limiting preventive care [17]. Despite the presence of a national oral health strategy, the overall investment in dental care remains low, and access to fluoride toothpaste is limited for much of the population [6,18].

Although prior research in Rwanda has documented oral health status and associated risk factors in children or select districts, there is limited evidence on the national prevalence and predictors of inadequate teeth cleaning practices among adults. Most existing studies are localized or qualitative in nature [1,2,9,10,13,17], and few use nationally representative data. Understanding the current patterns and predictors of non-daily teeth cleaning is vital to guide policy and preventive efforts. This study therefore aims to assess the prevalence and determinants of non-daily teeth cleaning practices among adults in Rwanda using data from the 2022 STEPS Survey.

## 2. Materials and Methods

This cross-sectional study analyzed data from the 2022 Rwanda NCD STEPS Survey, a nationally representative population-based survey targeting adults aged 18 to 69 years. The survey followed the World Health Organization’s Stepwise Approach to Surveillance, including Step 1 (behavioral risk factors), Step 2 (physical measurements), and Step 3 (biochemical assessments). For this study, data from Step 1—focused on self-reported oral hygiene behavior and related factors—were utilized.

A multi-stage cluster sampling strategy was applied. In the first stage, 400 enumeration areas (EAs) were selected from a national sampling frame using probability proportional to size. In the second stage, 15 households were randomly selected within each EA using systematic random sampling. Finally, in the third stage, one eligible adult aged 18–69 years was randomly selected from each sampled household using a tablet-based algorithm. Eligible participants were required to understand Kinyarwanda, English, or French. Individuals with mental, speech, or hearing disabilities that hindered effective communication were excluded.

The final survey sample comprised 5776 adults, with an overall response rate of 96.3%. Ethical approval was obtained from the Rwanda National Ethics Committee (Ref: 553/RNEC/2021), and all participants provided written informed consent prior to participation. The primary outcome for this analysis was teeth cleaning frequency, categorized into daily (once or twice a day) and non-daily (cleaning less than once daily or not at all). Independent variables included demographic, socioeconomic, and behavioral factors: age, sex, marital status, education, employment, income, residence, alcohol and tobacco use, physical inactivity, dental visits, and toothpaste use.

Weighted frequencies and 95% confidence intervals (CIs) were used to describe the distribution of tooth-cleaning behaviors. Binary logistic regression models were applied to assess associations between explanatory variables and non-daily teeth cleaning. First, Crude Odds Ratios (CORs) were estimated, followed by multivariable logistic regression to obtain Adjusted Odds Ratios (AORs) controlling for potential confounders. Associations were considered statistically significant if the 95% CI did not include 1. All the analysis was done using Stata 18 software.

## 3. Results

The sample for this study consisted of 5673 participants. The overall prevalence of non-daily teeth cleaning was 33.1% (95% CI: 31.0–35.2), aligning with the findings from the RBC (2022) [8]. A detailed breakdown of teeth cleaning frequency revealed that the highest proportion of participants, 47.6% (95% CI: 45.5–49.7), cleaned their teeth once a day, while 19.3% (95% CI: 17.6–21.2) reported teeth cleaning twice or more a day. A total of 16.2% (95% CI: 14.9–17.6) cleaned their teeth 2–6 times a week, and 7.9% (95% CI: 7.0–9.1) reported never cleaning their teeth. Smaller proportions of participants cleaned their teeth only once a week (4.4%, 95% CI: 3.8–5.1), 2–3 times a month (2.0%, 95% CI: 1.6–2.5), and once a month (2.4%, 95% CI: 2.0–3.0).

Among individuals who clean their teeth, the majority (88.0%) use a toothbrush, with a prevalence of 86.1% for toothpaste use as shown in Table 1. Wooden toothpicks are the second most common tool, used by 32.6% of respondents. Less frequently used methods include plastic toothpicks (1.0%), dental floss (1.8%), and chewsticks (1.6%). Charcoal is also used by 5.9% of individuals, while other methods account for 6.0%. This indicates that traditional and less conventional methods still play a role in oral hygiene among some Rwandans.

The analysis of non-daily teeth cleaning in Table 2 revealed notable differences across various factors. Non-daily teeth cleaning was more common among males (34.3%) than females (31.9%) and increased with age, from 25.0% among 18–29-year-olds to 51.0% among those aged 60–69. Non-daily teeth cleaning was lowest among single individuals (20.8%) and highest among separated individuals (44.0%). Rural residents (38.0%) reported significantly higher prevalence than their urban counterparts (11.2%).

Individuals with no formal education (48.1%) and low income (34.9%) had the highest prevalence of non-daily teeth cleaning. In contrast, those with higher education (10.0%) and higher income (17.9%) had notably lower rates. Unemployed individuals showed a lower prevalence (21.6%) than employed ones (36.0%).

Behavioral factors also played a role—smokers (53.7%) and alcohol users (39.0%) had higher prevalence of non-daily teeth cleaning than their counterparts. Interestingly, physically inactive individuals had a lower prevalence (25.8%) compared to those who were active (33.4%). Oral health practices also showed variation: individuals not using toothpaste had a much higher prevalence of non-daily teeth cleaning (44.6%) than those who did (24.5%). Recent dental visits were not strongly associated with cleaning frequency.

Table 3 shows the associations between various demographic, socioeconomic, and behavioral factors with non-daily teeth cleaning. Residence status was also significantly associated with teeth cleaning frequency. Rural residents had significantly higher odds of non-daily teeth cleaning compared to urban residents (COR = 4.9, 95% CI: 3.6–7.1), and this relationship remained significant after adjustment (AOR = 2.5, 95% CI: 1.5–4.1).

For educational status, individuals with basic education had lower odds of non-daily teeth cleaning compared to those with no formal education in the crude model (COR = 0.5, 95% CI: 0.4–0.6), but this association was not statistically significant after adjustment (AOR = 0.8, 95% CI: 0.7–1.0). Those with higher education had significantly lower odds of non-daily teeth cleaning compared to individuals with no formal education even after adjustment (COR = 0.1, 95% CI: 0.1–0.2; AOR = 0.3, 95% CI: 0.2–0.6).

Income status was another significant factor, with individuals earning less than or equal to USD 65 having higher odds of non-daily teeth cleaning compared to those earning more than USD 65 (COR = 2.5, 95% CI: 1.7–3.6), and this association remained significant after adjustment (AOR = 2.0, 95% CI: 1.3–3.2).

For oral health practices, individuals who did not use toothpaste had significantly higher odds of non-daily teeth cleaning compared to those who did (COR = 2.5, 95% CI: 2.0–3.0), and this effect remained significant after adjustment (AOR = 1.3, 95% CI: 1.0–1.7).

Table 3 indicates that several demographic and behavioral variables were not significantly associated with non-daily teeth cleaning in the adjusted analysis, despite some showing crude associations. For instance, age group, marital status, employment status, alcohol use, and smoking demonstrated significant associations in the unadjusted models. Older age groups had higher odds of non-daily teeth cleaning, and individuals who were married or separated had increased odds compared to singles. Similarly, smokers and alcohol users showed higher odds of non-daily teeth cleaning in the crude analysis. However, after adjusting for potential confounders, none of these associations remained statistically significant. Variables such as gender, physical inactivity, and dental visits were not significant in either model, suggesting that these factors may have limited influence on teeth cleaning frequency once socioeconomic and behavioral variables were accounted for.

## 4. Discussion, Recommendations, Limitations, and Future Research

### 4.1. Discussion

This study provides critical insights into the socioeconomic and behavioral factors associated with non-daily teeth cleaning among adults in Rwanda. While the majority reported daily teeth cleaning, a considerable proportion did not meet the recommended standard of twice-daily brushing. Some still relied on traditional methods such as chewsticks and charcoal. Although these alternatives are culturally accepted and commonly used, particularly in rural areas, studies have shown they are less effective in removing plaque and preventing caries and periodontal diseases compared to toothbrushes with fluoride toothpaste [19,20,21]. Similar trends have been reported in other low- and middle-income countries, where economic barriers and cultural practices contribute to continued reliance on traditional cleaning agents [20,22].

Rural residence emerged as a significant factor associated with non-daily teeth cleaning. Rural populations often face numerous challenges, including poor access to oral health facilities, lack of trained personnel, limited availability of dental hygiene products, and minimal exposure to oral health education campaigns [1,4,9,23,24,25]. Previous studies in Rwanda and elsewhere have consistently shown that urban residents are more likely to adopt modern oral hygiene practices, while rural residents depend on traditional tools and are less likely to afford or access fluoride toothpaste [4,6,20,22]. In countries such as Nigeria, Eritrea, and Uganda, rural dwellers have also reported lower usage of toothbrushes and toothpaste, often due to both cost and availability constraints [14,15,16,20,24].

Education level played a crucial role, with individuals having higher educational attainment significantly less likely to practice non-daily teeth cleaning. This supports findings from Rwanda and other African countries that link education with improved oral health literacy, better understanding of recommended practices, and a greater likelihood of seeking preventive care [3,4,9,11,25,26]. Oral hygiene knowledge influences behavior, and those with secondary or tertiary education are more likely to brush twice daily and use toothpaste, especially fluoride-containing types, as seen in studies from Kenya, Mauritius, and Ethiopia [20,25,27].

Income status was also significantly associated with brushing frequency. Those in lower-income brackets had higher odds of non-daily teeth cleaning, likely due to financial barriers limiting their ability to purchase toothbrushes and toothpaste regularly [3,4,8,22]. In Rwanda, where a large portion of the population remains in the lower wealth categories, this economic factor plays a central role in shaping hygiene behaviors. Studies from other LMICs affirm that affordability challenges are a common reason for poor oral hygiene, even when individuals understand its importance [20,28]. Moreover, studies have highlighted that those from lower-income households tend to delay or avoid dental visits until there is pain or infection, further reducing their exposure to professional oral hygiene advice [13,22].

Toothpaste use was another significant behavioral factor in this study. Respondents who used toothpaste were more likely to brush daily. This aligns with previous findings from Rwanda and other countries that emphasize the role of fluoride toothpaste in promoting regular brushing and preventing dental diseases [4,6,13,19]. The effectiveness of fluoride in preventing caries is well established, and daily use remains the most cost-effective preventive intervention available [19,21,27]. However, toothpaste cost and distribution gaps mean many rural and low-income households either use it infrequently or substitute with ash, salt, or herbal mixtures, which have limited preventive benefits [20,25,28]. In Eritrea, for example, a large number of students reported not knowing whether their toothpaste contained fluoride, reflecting both access and awareness issues [20].

Together, these findings illustrate the complex interplay between social, economic, and behavioral factors in influencing oral hygiene behavior in Rwanda. While knowledge and attitudes play a role, the lack of infrastructure, limited oral health promotion, and economic constraints remain major barriers to improving population-wide oral hygiene. As noted in similar studies, interventions must go beyond individual behavior change and address the broader structural determinants of health [24,27,28,29].

### 4.2. Recommendations

This study highlights the need for targeted interventions to improve oral hygiene practices in Rwanda, particularly among rural, low-income, and less-educated populations. While the Rwanda Ministry of Health (2019) [18] report plan and other ongoing initiatives have laid a foundation, the findings of this study suggest several areas for enhancement and expansion:

1. Increase awareness and knowledge enhancement of Oral Health:

Limited information on oral health and its connection to overall well-being contributes to the prevalence of non-daily teeth cleaning behaviors. To address this, it is essential to leverage media and social media platforms to raise public awareness and enhance knowledge about the importance of oral hygiene in preventing oral diseases. By delivering targeted, accessible educational content through these channels, the population can be better informed about the critical role oral health plays in maintaining general health, thereby promoting consistent oral hygiene practices.

2. Enhancing Rural Oral Health Outreach and Mobile Dental Clinics:

Rural residents were found to have significantly higher odds of non-daily teeth cleaning. Expanding rural outreach programs, including mobile dental clinics, would provide access to underserved communities, offering oral health education and affordable dental products such as toothbrushes and toothpaste. These mobile services can integrate oral health with other community health services, ensuring regular visits to rural areas and offering fluoride treatments, screenings, and education on proper dental hygiene.

3. Culturally Sensitive Health Education Campaigns:

Traditional oral hygiene methods such as chewsticks and charcoal are still used by some individuals, particularly in rural areas. Instead of discouraging these practices outright, culturally sensitive campaigns should be developed that educate people on the benefits of modern dental products while recognizing the role of traditional practices. This approach can help gradually shift communities toward using more effective methods without alienating their cultural heritage. Public health messaging should emphasize the importance of using fluoride toothpaste for cavity prevention and explore ways to integrate traditional methods with modern practices, such as promoting the use of toothbrushes alongside chewsticks.

4. Improving Access to Affordable Dental Products in Low-Income Communities:

Lower-income individuals were significantly more likely to engage in non-daily teeth cleaning. To address this, programs that subsidize toothpaste and toothbrushes for low-income households should be expanded. Initiatives could also include partnerships with local manufacturers to produce affordable, locally made dental products. The distribution of free or low-cost dental products through community health centers and schools can help overcome financial barriers and encourage daily teeth cleaning.

5. Strengthening School-Based Oral Health Programs:

The existing school-based oral health programs have been successful in promoting good oral hygiene habits among children. Expanding these programs to include more rural schools and incorporating supervised teeth cleaning activities that include the use of toothpaste can help instill lifelong habits. Schools can also serve as distribution points for free toothbrushes and toothpaste, particularly for children from low-income families, ensuring they have the necessary tools for maintaining good oral hygiene.

6. Integrating Oral Health into Community Health Programs:

Oral health should be integrated into broader community health programs, especially those focused on non-communicable diseases (NCDs). Community health workers can be trained to deliver oral health education, distribute dental products, and refer individuals for dental care as part of their regular health visits. Community health campaigns can promote the use of fluoride toothpaste and educate the public on the importance of daily teeth cleaning while also addressing misconceptions about traditional practices.

7. Targeting Vulnerable Populations for Improved Access

Vulnerable groups, such as individuals with lower education levels and those in rural areas, require targeted interventions. Community health campaigns and services tailored to these groups should focus on improving oral health literacy, with clear messaging on the importance of regular teeth cleaning and the benefits of using modern dental products. Providing these groups with easier access to affordable or free dental products will encourage consistent teeth cleaning habits.

### 4.3. Limitations

The study has several limitations that should be acknowledged. First, its cross-sectional design limits the ability to establish causality between socioeconomic factors and oral hygiene behaviors. Additionally, the reliance on self-reported data may introduce recall bias, potentially affecting the accuracy of the responses. Another limitation is the possible underrepresentation of vulnerable groups, such as individuals with disabilities, who may not be fully captured in the data.

### 4.4. Future Research

Future research should focus on exploring the health impacts of traditional oral hygiene methods, such as chewsticks and charcoal, which remain common in certain communities. Longitudinal studies are also necessary to track changes in oral hygiene behavior over time, providing valuable insights into the effectiveness of interventions. Further investigation is needed to understand the specific barriers to accessing dental products in low-income communities. Finally, there is a need to evaluate the impact of Rwanda’s National Oral Health Strategic Plan, to identify areas that require further improvement and ensure that policies effectively promote oral health across the population.

## 5. Conclusions

This study has revealed important socioeconomic and behavioral factors that contribute to non-daily teeth cleaning among adults in Rwanda. Rural residence, lower education levels, lower income, and the non-use of toothpaste emerged as the strongest predictors of inadequate oral hygiene practices. These findings highlight the disparities in oral hygiene behaviors, with rural and low-income populations facing greater challenges in maintaining regular teeth cleaning. The persistence of traditional methods like chewsticks and charcoal, especially in rural areas, points to the influence of cultural practices on oral health behaviors. While these practices remain widespread, efforts to integrate modern dental products like toothpaste into daily routines are critical for improving oral health outcomes.

Addressing these challenges requires targeted public health interventions that focus on improving access to dental care products, enhancing education, and reaching underserved populations. By leveraging Rwanda’s existing oral health programs and expanding on them, these interventions can play a pivotal role in reducing the burden of oral diseases across the country. Future research should continue to explore the long-term effectiveness of these interventions and investigate how traditional oral hygiene practices might be adapted or integrated into modern health initiatives.

## Figures and Tables

**Table 1:** Prevalence of different teeth cleaning methods.

	Teeth Cleaning		
	Frequency	Prevalence (%)	95% CI
Toothbrush	4372	88.0	86.5–89.4
Wooden toothpicks	1809	32.6	30.6–34.6
Plastic toothpicks	60	1.0	0.7–1.5
Thread (dental floss)	73	1.8	1.3–2.5
Charcoal	322	5.9	5.1–6.9
Chewstick/miswak	63	1.6	1.1–2.3
Other	347	6.0	5.1–7.1
Toothpaste	4228	86.1	84.6–87.5

**Table 2:** Frequencies and prevalence of non-daily teeth cleaning by demographic, socioeconomic, behavioral, and oral health practice factors.

	Non-Daily Teeth Cleaning		
	Frequency	Prevalence (%)	95% CI
Demographic Factors			
Sex			
Male	2128	34.3	31.4–37.2
Female	3545	31.9	29.5–34.3
Age group			
18–29	1310	25.0	21.9–28.0
30–44	2383	36.7	34.2–39.1
45–59	1293	41.4	37.8–45.0
60–69	687	51.0	46.3–55.8
Marital status			
Single	937	20.8	17.3–24.4
Married	3636	37.9	35.5–40.3
Separated	1095	44.0	39.9–48.2
Residence status			
Urban	1141	11.2	7.6–14.7
Rural	4532	38.0	35.7–40.3
Socioeconomic factors			
Educational status			
No Formal education	2030	48.1	45.1–51.0
Basic education	2793	32.6	29.9–35.2
Higher education	820	10.0	7.0–13.1
Employment status			
Employed	4829	36.0	33.9–38.1
Unemployed	844	21.6	17.6–25.6
Income status in USD			
>48.8	419	17.9	12.4–23.4
≤48.8	4792	34.9	32.7–37.1
Behavioral factors			
Alcohol use			
No	488	31.1	25.7–36.5
Yes	2754	39.0	36.3–41.7
Smoking			
No	5169	31.5	29.4–33.7
Yes	504	53.7	48.3–59.1
Physical inactivity			
No	5412	33.4	31.4–35.5
Yes	261	25.8	17.5–34.1
Oral health practices			
Dental visit			
<6 Months	343	36.1	28.8–43.4
≥6 Months	5330	32.9	30.8–35.1
Use of toothpaste			
Yes	4228	24.5	22.5–26.5
No	879	44.6	39.9–49.2

**Table 3:** Crude and adjusted odds ratios with 95% confidence intervals for non-daily teeth cleaning by demographic, socioeconomic, behavioral, and oral health factors.

	Crude Odds Ratio (COR) [95% CI]	Adjusted Odds Ratio (AOR) [95% CI]
Demographic Factors		
Gender		
Male	Reference	Reference
Female	0.9 [0.8–1.0}	0.9 [0.7–1.1}
Age group		
18–29	Reference	Reference
30–44	1.7 [1.5–2.1}	0.9 [0.7–1.4}
45–59	2.1 [1.7–2.6}	1.0 [0.7–1.5}
60–69	3.1 [2.5–3.9}	1.3 [0.8–2.0}
Marital status		
Single	Reference	Reference
Married	2.3 [1.8–2.9}	1.5 [0.9–2.4}
Separated	3.0 [2.3–3.9}	1.6 [0.9–2.7}
Residence status		
Urban	Reference	Reference
Rural	4.9 [3.6–7.1}	2.5 [1.5–4.1}
Socioeconomic factors		
Educational status		
No Formal education	Reference	Reference
Basic education	0.5 [0.4–0.6}	0.8 [0.7–1.0}
Higher education	0.1 [0.1–0.2}	0.3 [0.2–0.6}
Employment status		
Employed	Reference	Reference
Unemployed	0.5 [0.4–0.6}	1.0 [0.7–1.7}
Income status in USD		
>48.8	Reference	Reference
≤48.8	2.5 [1.7–3.6}	2.0 [1.3–3.2}
Behavioral factors		
Alcohol use		
No	Reference	Reference
Yes	1.4 [1.1–1.8}	1.3 [0.9–2.0}
Smoking		
No	Reference	Reference
Yes	2.5 [2.0–3.2}	1.2 [0.9–1.7}
Physical inactivity		
No	Reference	Reference
Yes	0.7 [0.5–1.1}	0.7 [0.4–1.3}
Oral health practices		
Dental visit		
<6 Months	Reference	Reference
≥6 Months	0.9 [0.6–1.2}	0.9 [0.5–1.5}
Use of toothpaste		
Yes	Reference	Reference
No	2.5 [2.0–3.0}	1.3 [1.0–1.7}

## Data Availability

The datasets used and analyzed during the current study are available from the corresponding author upon reasonable request. The data are not publicly available due to privacy reasons.

## Abbreviations

The following abbreviations are used in this manuscript:

RNEC	Rwanda National Ethics Committee
RBC	Rwanda Biomedical Centre
COR	Crude odds ratio
AOR	Adjusted odds ratio
NCD	Non-communicable disease
CI	Confidence interval
